# Pleural and pericardial effusions as prognostic factors in patients with acute pulmonary embolism: a multicenter study

**DOI:** 10.1007/s10140-024-02281-7

**Published:** 2024-08-30

**Authors:** Hans-Jonas Meyer, Constantin Ehrengut, Anar Aghayev, Mattes Hinnerichs, Dominik Schramm, Felix G. Meinel, Jan Borggrefe, Alexey Surov

**Affiliations:** 1 Department of Diagnostic and Interventional Radiology, University of Leipzig, Leipzig, Germany; 2 Department of Radiology and Nuclear Medicine, Otto von Guericke University, Magdeburg, Germany; 3 Department of Diagnostic and Interventional Radiology, University of Halle- Wittenberg, Halle (Saale), Germany; 4 Department of Diagnostic and Interventional Radiology, University of Rostock, Rostock, Germany; 5 Department of Radiology, Neuroradiology and Nuclear Medicine, Johannes Wesling University Hospital, Ruhr University Bochum, Minden, Germany

**Keywords:** CT, Pleural effusion, Acute pulmonary embolism

## Abstract

**Purpose:**

The prognostic role of pleural and pericardial effusion in patients with acute pulmonary embolism (PE) is still unclear with a trend for worse clinical outcome. The aim of the present study was to demonstrate the prognostic role of pleural and pericardial effusion in patients with acute PE in a large multicentre setting.

**Methods:**

The investigated patient sampled was retrospectively comprised of 1082 patients (494 female, 45.7%) with a mean age of 63.8 years ± 15.8. In every case, contrast enhanced computed tomography (CT) pulmonalis angiography was analyzed to diagnose and quantify the pleural and pericardial effusion. The 30-day mortality was the primary endpoint of this study.

**Results:**

A total of 127 patients (11.7%) died within the 30-day observation period. Pleural effusion was identified in 438 patients (40.5%) and pericardial effusion was identified in 196 patients (18.1%). The presence of pleural effusion was associated with 30-day mortality, HR = 2.78 (95%CI1.89-4.0), *p* < 0.001 (univariable analysis), and HR = 2.52 (95%CI1.69-3.76), *p* < 0.001 (multivariable analysis). The pleural effusion width and density were not associated with 30-day mortality. The presence of pericardial effusion was not associated with 30-day mortality in multivariable analysis, HR = 1.28 (95%CI 0.80–2.03), *p* = 0.29.

**Conclusions:**

Pleural effusion is a common finding in patients with acute pulmonary embolism, occurring in 40.5% of cases, and is a prognostic imaging finding associated with 30-day mortality. The presence of pleural effusion alone, regardless of volume or density, has been shown to be prognostic and should be included in CT reports. The prognostic role of pericardial effusion is limited.

## Introduction

Acute pulmonary embolism (PE) is a potentially life-threatening cardiovascular disease with 30-day mortality rates ranging from 0.5% to over 20%, depending on clinical symptoms at presentation [1–3]. However, there are also low-risk clinical presentations without serious complications. Therefore, an immediate risk stratification of patients with acute PE at time of presentation is crucial for patient care.

The CT pulmonary angiography (CTPA) has been established as the diagnostic clinical gold standard for the diagnosis of PE with a reported sensitivity and specificity of up to 100% [4, 5]. It is the first imaging performed in these patients, most commonly directly after the admission to the hospital. Therefore, risk stratification based on CTPA is important [5, 6].

Contrast reflux into the inferior vena cava and right ventricular strain have been described as important prognostic CT signs [6, 7]. However, there is a definite need to further utilize the CTPA images for prognostic stratification in PE.

Pleural effusions occur in 30–50% of patients with PE [8]. Although frequently observed in patients with PE, the exact prevalence of pleural effusion is difficult to determine due to heterogeneous published results [8]. However, PE is a common cause of pleural effusion, ranking fourth after congestive heart failure, cancer, and pneumonia [9]. Although it is now clear that pleural effusion has a high incidence in patients with PE and is closely associated with the prognosis of PE, the results of recent studies investigating the prognostic relevance of pleural effusion are still inconclusive [10, 11]. The prognostic role of the presence of PE was demonstrated in a recent meta-analysis of 13,430 patients with a reported relative risk of 2.19 (95% CI: 1.53–3.15, *p* < 0.001) for 30-day mortality [11]. However, little is known about the CT-derived features of pleural effusion, namely CT density and width of pleural effusion, which may add prognostic information to the mere presence of effusion. Clearly, it is important to understand the clinical correlations between pleural effusion and mortality in PE patients in order to appropriately stratify and guide the management of PE patients.

Pericardial effusion is even rarer in patients with PE, with a reported incidence of 7% [12]. There may be some prognostic relevance for the occurrence of pericardial effusion, but published data are sparse compared to pleural effusion [12].

Therefore, the rationale of the present multicentric analysis was to elucidate the prognostic relevance of the presence of pleural and pericardial effusion and the quantified assessment of the effusions as prognostic feature in patients with acute PE.

## Methods

This retrospective study was approved by the institutional review Board (number 20–719). *blinded.

Inclusion criteria for the present study.

- diagnosis of acute PE;
- pretreatment contrast enhanced CT examination of diagnostic image quality in pulmonary artery phase;
- complementary clinical parameters including outcome: 30-day mortality.

Exclusion criteria were:

- missing or incomplete documentation of clinical parameters;
- non-diagnostic image quality of CT studies;
- chronic PE;
- primary/secondary malignant diseases with affection of the pleura or pericardium;

### Clinical features

The following clinical parameters were retrieved at the time-point of hospital admission:

- relevant clinical comorbidities (active malignant disease, surgery performed within the last 4 weeks, chronic-lung disease, chronic heart failure).
- blood pressure (mmHg), heart rate (n/minute).
- the simplified pulmonary embolism severity index (sPESI) score was calculated as proposed by the publication [13]. In short, the score can range from 0 to 6 points. It comprises the items, age, cancer disease, chronic heart failure, heart frequency, systolic blood pressure, oxygen saturation.
- Mortality, assessed in days after diagnosis of PE.
- the primary study end-point was all cause 30-day mortality.

### Effusion imaging analysis

All measurements were taken manually by experienced radiologists in the three centers using the clinical reporting and viewing software.

**Fig. 1:**
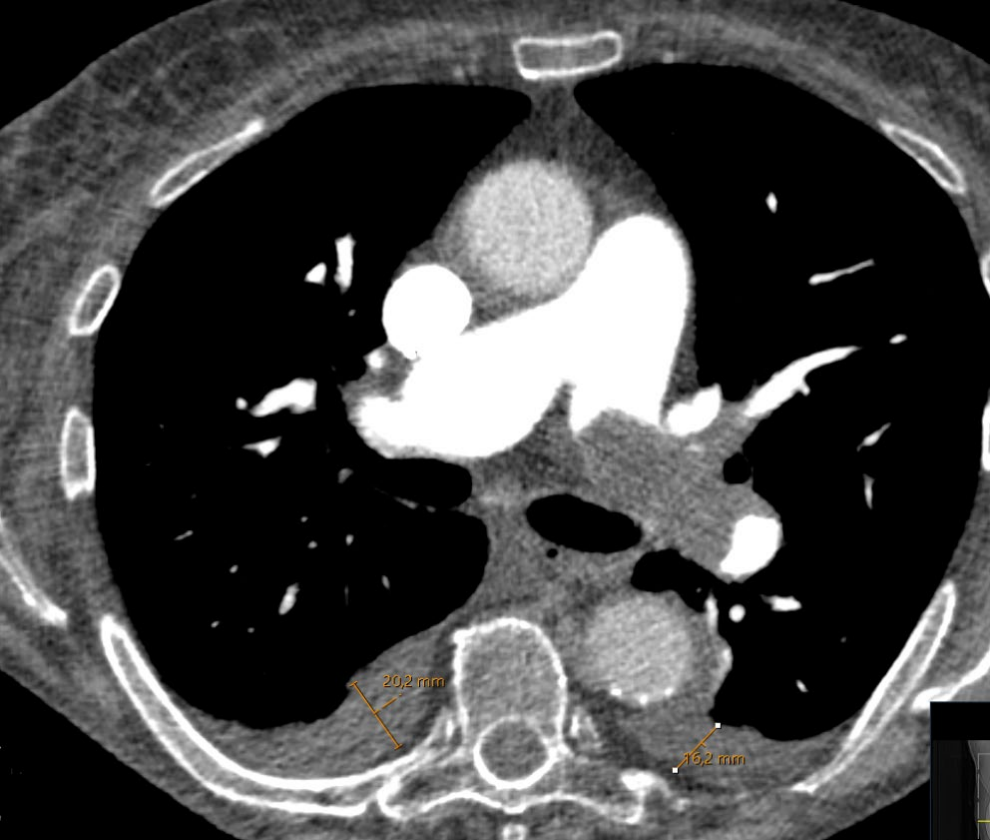
A representative case of the patient sample with acute central PE and pleural effusions. The patient survived within the 30-day observation period. One can appreciate the pleural effusion on both sides. On the right side it measures 20.2 mm and on the left side 16.2 mm

All readers were blinded to clinical outcomes, as the imaging analysis was performed by another researcher than the extraction of the clinical data from the patient files. The presence of pleural effusion was diagnosed according to the definition by the American College of Radiology [14]: the presence of > 3 mm fluid within the pleural space was considered abnormal. The width of pleural effusion was measured as the largest distance in axial CT reconstruction, with a medium soft (“soft tissue”) convolution kernel (Fig. 1). The axial slice was used, which demonstrated the greatest width of the pleural effusion. Density of pleural effusion was measured in a region of interest placed centrally within the effusion. In cases with bilateral pleural effusion, the largest width was used for the patient.

The presence of pericardial effusion, width and density was measured in the same manner. The criteria of the pericardial effusion is defined by the American College of Radiology with a width of 3 mm as a cut-off value [14].

### Statistical analysis

Statistical analysis was performed using SPSS (IBM SPSS Statistics for Windows. version 225.0: IBM corporation). Data were analyzed by means of descriptive statistics (absolute and relative frequencies). Spearman’s correlation coefficient (r) was used to analyze associations between investigated effusion parameters with clinical features after testing for normality distribution (Kolmogorov–Smirnov test). The investigated group differences (survivors vs. non-survivors) were calculated with Mann–Whitney-u test for comparisons of non-normally distributed, at least ordinally scaled parameters in unpaired samples and the Fisher’s exact test was used for categorical variables. Univariable binary logistic regression analysis was performed to investigate the associations between pleural and pericardial effusion values and 30-day mortality. In the next step, the statistically significant parameters were further analyzed in multivariable logistic regression analysis to adjust for potential confounders. In all instances, p-values below 0.05 were used to indicate statistical significance.

## Results

Overall, the patient sample comprised 1082 patients (494 female, 45.7%) with a mean age of 63.8 years ± 15.8 (range 18–82 years).

A total of 127 patients (11.7%) died within the 30-day observation period. Pleural effusion was identified in 438 patients (40.5%) and pericardial effusion was identified in 196 patients (18.1%). The mean width of pleural effusion was 28.9 mm ± 24.9 and the mean density was 9.2 HU ± 7.0. Regarding pericardial effusion the mean width was 10.0 mm ± 6.3 and the mean density was 12.7 HU ± 10.4. The mean sPESI score was 1.5 ± 1.1.

### Associations with sPESI score

**Fig. 2:**
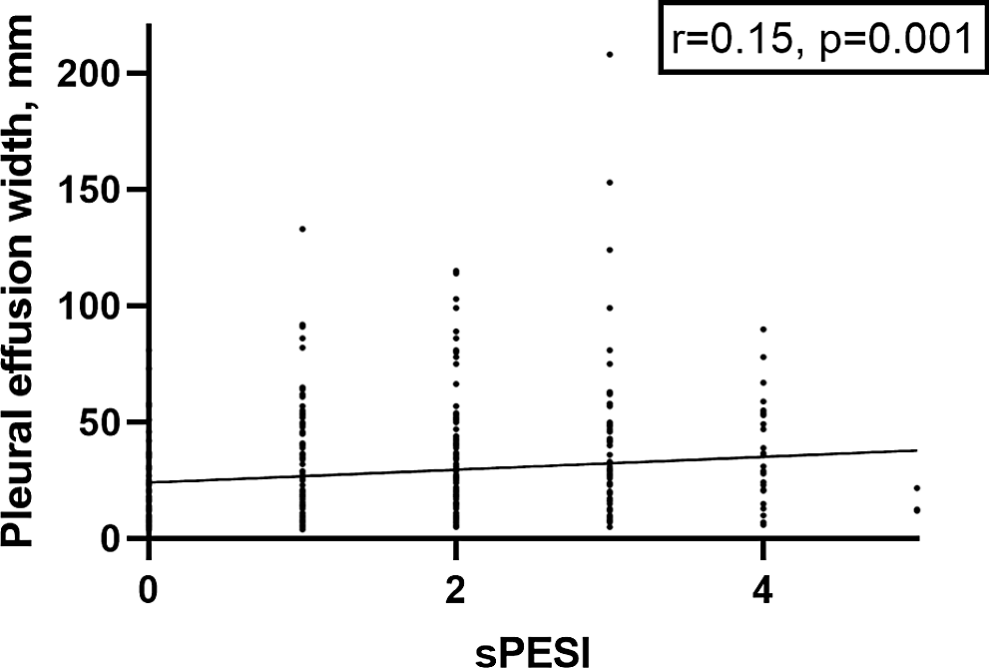
Spearman’s correlation analysis between sPESI score and pleural effusion width (*r* = 0.15, *p* = 0.001)

A weak positive association was identified between the pleural effusion width with sPESI score (*r* = 0.15, *p* = 0.001) (Fig. 2). For the other effusion parameters there were no statistically significant associations (*r*=-0.14, *p* = 0.06 for pericardial effusion width, *r* = 0.12, *p* = 0.08 for pericardial effusion density, *r* = 0.08, *p* = 0.10 for pleural effusion density, respectively).

**Fig. 3:**
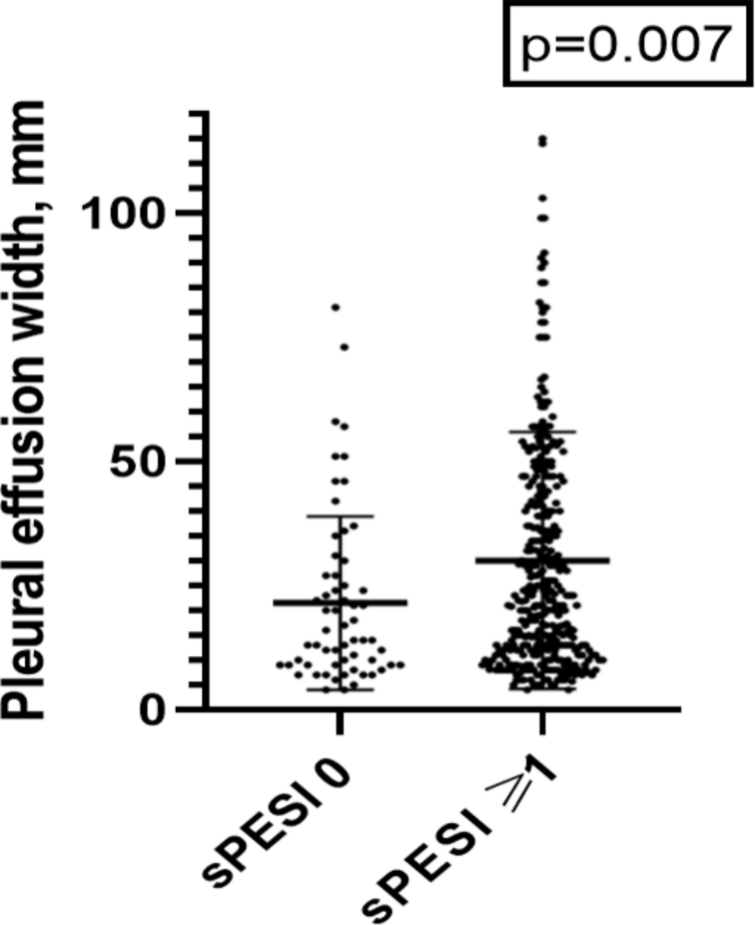
Comparison of the pleural effusion between cases with sPESI score 0 and ≥ 1. The width was significantly higher in cases with sPESI ≥ 1 (*p* = 0.007)

As a next step, we divided the patient sample accordingly to the proposed clinical threshold value of ≥ 1 for the sPESI score. There were 197 patients (18.2%) with a sPESI score 0 and 885 patients (81.8%) with ≥ 1. Pleural effusion occurred in 62 cases with sPESI score 0 (31.4%) and in 359 cases with sPESI ≥ 1 (40.6%), *p* = 0.11, pericardial effusion occurred in 24 of cases with sPESI score 0 (12.1%), and in 172 cases (19.4%) with sPESI ≥ 1, *p* = 0.05. The width of the pleural effusion was significantly larger in cases with sPESI ≥ 1 compared to sPESI 0 (*p* = 0.007), Fig. 3a. The density of the pleural effusion was also higher in cases with sPESI ≥ 1 (*p* = 0.04).

**Fig. 4:**
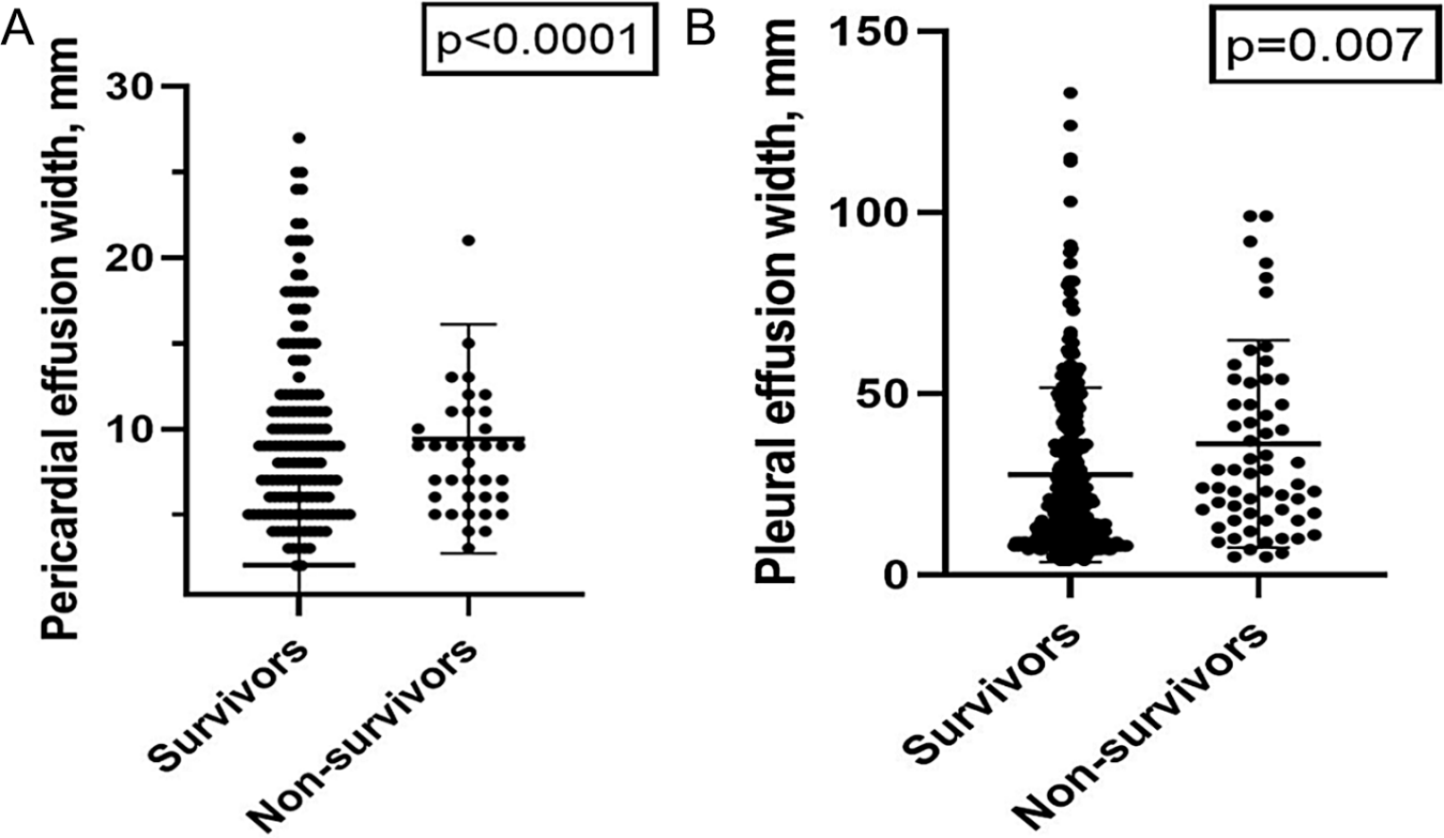
(**A**). Comparison of the pleural effusion width between survivors and non-survivors. The width was higher in non-survivors (*p* = 0.007). (**B**). Comparison of the pericardial effusion width between survivors and non-survivors. The width was higher in non-survivors (*p* < 0.0001)

Regarding pericardial effusion, the width was higher not statistically significant different (*p* = 0.08), whereas the density was higher in cases with sPESI ≥ 1 (*p* < 0.0001).

### Discrimination analysis

Pleural effusion occurred more frequently in cases with fatal outcome, 48.0% vs. 39.6%, but was not statistically significant, p=0.26.

The frequency of pericardial effusion was 29.1% in fatal cases compared with 16.7% in non-fatal cases, *p* = 0.01. Regarding the width, it was both larger in patients with fetal outcome for pleural and pericardial effusion (*p* = 0.007 and *p* < 0.0001, respectively, Fig. 4). For the density, the pericardial effusion showed higher HU values in fatal cases (*p* = 0.007), whereas it was not different for pleural effusion (*p* = 0.39). The sPESI score was significant higher in non-survivors compared to survivors (2.0 ± 1.1 versus 1.5 ± 1.1, *p* < 0.0001). The results are presented in Table 1.

**Table 1:** Pleural and pericardial effusion features accordingly to survivors and non-survivors

Effusion features	Survivors (*n* = 951)	Non-survivors (*n* = 127)	*P*-values
Presence of pleural effusion	377 (39.6%)	61 (48.0%)	0.26
Pleural effusion width (mm)	27.6 ± 24.1	36.2 ± 28.6	0.007
Pleural effusion density (HU)	9.1 ± 7.0	9.7 ± 6.8	0.39
Presence of pericardial effusion	159 (16.7%)	37 (29.1%)	0.01
Pericardial effusion width (mm)	2.0 ± 4.8	9.1 ± 6.7	< 0.0001
Pericardial effusion density (HU)	11.4 ± 9.2	17.6 ± 13.1	0.007
sPESI score	1.5 ± 1.1	2.0 ± 1.1	< 0.0001

*Abbreviations* simplified Pulmonary Embolism severity index, HU Hounsfield Unit

### Prediction of 30-day mortality

The results of the logistic regression analysis to predict 30-day mortality is presented in Table 2. The presence of pleural effusion was associated with 30-day mortality: HR = 2.78, 95%CI(1.89-4.0), *p* < 0.001 (univariable analysis) and HR = 2.52, 95%CI(1.69–3.76), *p* < 0.001 (multivariable analysis). The pleural effusion width and density were not associated with 30-day mortality. The presence of pericardial effusion was associated with 30-day mortality in univariable analysis, HR = 1.56, (95%CI1.01-2.43, *p* = 0.04) but could not reach statistical significance in the multivariable analysis, HR = 1.28, 95%CI(0.80–2.03), *p* = 0.29.

**Table 2:** Uni- and multivariable logistic regression analysis for 30-day mortality

Effusion features	Univariable HR	95%CI	*p*-value	Multivariable HR	95%CI	*p*-value
Presence of pleural effusion	2.78	1.89-4.0	< 0.001	2.52	1.69–3.76	< 0.001
Pleural effusion width	1.008	0.99–1.01	0.07			
Pleural effusion density	1.0	0.97–1.03	0.97			
Presence of pericardial effusion	1.56	1.01–2.43	0.04	1.28	0.80–2.03	0.29
Pericardial effusion width	1.04	1.01–1.07	0.01	1.02	0.99–1.06	0.15
Pericardial effusion density	1.004	0.97–1.03	0.79			
sPESI score	1.49	1.27–1.74	< 0.001	1.36	1.15–1.61	< 0.001
Age	1.01	1.007–1.03	0.003	1.01	0.99–1.02	0.15
Gender	0.71	0.49–1.05	0.08			

## Discussion

This study investigated the prognostic relevance of pleural and pericardial effusion in patients with acute PE in a German multicenter study. The main finding is that the presence of pleural effusion alone is a prognostic factor in patients with acute PE. The present analysis is one of the largest to date on this topic.

Acute PE is associated with a significant mortality, with reported short-term mortality of up to 20% [1–4]. The most established prognostic factor is systolic blood pressure [15, 16]. Risk stratification is also mainly based on systolic blood pressure [1]. Other important aspects include cardiac injury with blood parameters, age over 70 years, history of bed rest for more than five days, cancer, chronic obstructive pulmonary disease, renal failure, heart failure, cardiovascular disease, and tachycardia [1, 16].

Risk stratification of acute PE is very important for treatment planning. Patients with low-risk PE can be treated with anticoagulation in most cases, whereas patients with severe PE may require mechanical revascularization [17].

An important aspect of the present analysis is that only the presence of pleural effusion carries prognostic information, whereas quantification by width or density does not carry additional relevant information. At first glance, this seems to contradict clinical routine. Possible confounding factors could be that the volume of the pleural or pericardial effusion is highly variable during the course of the patient’s illness. Moreover, both may be confounded by previous drainage treatment, which could not be accounted for in the present analysis. The exact timing of CT seems to be crucial for correct volumetry and Hounsfield measurement, which may be too heterogeneous in the present cohort.

Nevertheless, this result of the present analysis is of interest because it only reports the presence, which could be easily performed in clinical routine without the need for more complex measurements by the radiologist.

Pleural effusion is very common in critically ill patients with various causes, including viral pleuritis, congestive heart failure, or cancer [9]. Notably, acute PE is the fourth most common cause of pleural effusion after congestive heart failure, cancer, and pneumonia [9].

The prognostic role of the presence of pleural effusion was demonstrated in a recent meta-analysis of 13,430 patients with a reported relative risk of 2.19 (95% CI: 1.53–3.15, *p* < 0.001) for 30-day mortality [11]. Our present data are very well in line with this previous study. It is important to consider that only the presence of pleural effusion and not the quantification of pleural effusion was used in the meta-analysis. Second, pleural effusion was also measured on other imaging modalities, which could lead to higher heterogeneity of results. CT can be considered as the most standardized imaging modality, although sonography also has a high sensitivity for the diagnosis of pleural effusion [18].

In contrast to pleural effusion, the prognostic role of pericardial effusion in patients with acute PE is less well studied in the literature [12].

A study from Turkey evaluated 570 patients with acute PE. The incidence of pericardial effusion in this study was 7%, which is significantly lower compared to the present results. They showed a statistical signal for the prognostic relevance of pericardial effusion (*p* = 0.004), but it did not reach statistical significance in multivariate analysis [12].

Previously, the presence of pleural and pericardial effusion was incorporated into a score system, and both contributed equally to the score [19]. This constructed score was more accurate than the clinical score sPESI (AUC of 0.82 vs. 0.75) in the studied cohort of 1698 cases. In this study, the frequency of pericardial effusion was 21.7% in the survivor group compared to 40% in the non-survivor group (*p* < 0.001), which is slightly higher than in the present cohort [19].

In a meta-analysis of prognostic signs on CT images, right heart dilatation was the only statistically relevant finding with a reported 2.5-fold risk of all-cause mortality [6]. Notably, despite the complexity of the methodology, thrombus burden has been investigated in several studies with inconclusive results [20, 21]. Therefore, it remains a challenge to define prognostically relevant CT findings in acute PE.

An important aspect of the present analysis is that the hazard ratio for the presence of pleural effusion is even higher than that of the most commonly used sPESI score. It is debatable whether the addition of pleural effusion could improve the diagnostic accuracy of this score.

Several limitations of the present study need to be addressed. First, it is a retrospective study from different centers located in Germany. Second, although the measurements performed can be considered reliable, the possibility of some reader bias should be considered. Especially since no central reading of the CT images was performed. Third, there might be some confounding factors induced by previous drainage treatment, which could influence the width of the effusions. Fourth, we could not adjust for other causes of pleural effusion, such as congestive heart failure, which might introduce some confounding bias into the analysis. In addition, there could be some bias introduced by infarct pneumonia, which could also cause pleural effusion. However, only a few patients with pneumonia were identified in the current patient sample and further subgroup analysis was not possible.

## Conclusions

Pleural effusion is a common finding in patients with acute pulmonary embolism, occurring in 40.5% of cases, and is a prognostic imaging finding associated with 30-day mortality. The presence of pleural effusion alone, regardless of volume or density, has been shown to be prognostic and should be included in CT reports. The prognostic role of pericardial effusion is limited.
